# The Efficacy of Individualized, Community-Based Physical Activity to Aid Smoking Cessation: A Randomized Controlled Trial

**DOI:** 10.1155/2023/5535832

**Published:** 2023-05-26

**Authors:** Michelle B. Stockton, Kenneth D. Ward, Barbara S. McClanahan, Mark W. Vander Weg, Mace Coday, Nancy Wilson, George Relyea, Mary C. Read, Stephanie Connelly, Karen C. Johnson

**Affiliations:** ^1^College of Health Sciences, The University of Memphis, Memphis, TN, USA; ^2^School of Public Health, The University of Memphis, Memphis, TN, USA; ^3^Departments of Community and Behavioral Health, Internal Medicine, and Psychological and Brain Sciences, University of Iowa, Iowa City, IA and Iowa City VA Health Care System, Iowa City, IA, USA; ^4^Department of Preventive Medicine, The University of Tennessee Health Science Center, Memphis, TN, USA; ^5^Methodist Healthcare, Memphis, TN, USA

## Abstract

**Objective:**

The efficacy of individualized, community-based physical activity as an adjunctive smoking cessation treatment to enhance long-term smoking cessation rates was evaluated for the Lifestyle Enhancement Program (LEAP).

**Methods:**

The study was a two-arm, parallel-group, randomized controlled trial. All participants (*n* = 392) received cessation counseling and a nicotine patch and were randomized to physical activity (*n* = 199; YMCA membership and personalized exercise programming from a health coach) or an equal contact frequency wellness curriculum (*n* = 193). Physical activity treatment was individualized and flexible (with each participant selecting types of activities and intensity levels and being encouraged to exercise at the YMCA and at home, as well as to use “lifestyle” activity). The primary outcome (biochemically verified prolonged abstinence at 7-weeks (end of treatment) and 6- and 12-months postcessation) and secondary outcomes (7-day point prevalent tobacco abstinence (PPA), total minutes per week of leisure time physical activity and strength training) were assessed at baseline, 7 weeks, 6 months, and 12 months.

**Results:**

Prolonged abstinence in the physical activity and wellness groups was 19.6% and 25.4%, respectively, at 7-weeks, 15.1% and 16.6% at 6-months, and 14.1% and 17.1% at 12 months (all between-group *P* values >0.18). Similarly, PPA rates did not differ significantly between groups at any follow-up. Change from baseline leisure-time activity plus strength training increased significantly in the physical activity group at 7 weeks (*P* = 0.04). Across treatment groups, an increase in the number of minutes per week in strength training from baseline to 7 weeks predicted prolonged abstinence at 12 months (*P* ≤ 0.001). Further analyses revealed that social support, fewer years smoked, and less temptation to smoke were associated with prolonged abstinence over 12 months in both groups.

**Conclusions:**

Community-based physical activity programming, delivered as adjunctive treatment with behavioral/pharmacological cessation treatment, did not improve long-term quit rates compared to adjunctive wellness counseling plus behavioral/pharmacological cessation treatment. This trial is registered with https://beta.clinicaltrials.gov/study/NCT00403312, registration no. NCT00403312.

## 1. Introduction

Despite substantial progress in reducing tobacco use in the United States, 12.5% of adults continue to smoke cigarettes, accounting for over 480,000 deaths every year [1]. Given this substantial burden, improving smoking cessation strategies remains a priority. The most effective cessation treatments combine behavioral and pharmacological support, but even intensive treatments (e.g., multiple counseling sessions, and/or medications) rarely achieve long-term quit rates above 25% [2, 3].

Physical activity has emerged as a potential strategy to enhance cessation outcomes. Acute bouts of various forms of exercise (e.g., isometric activity such as muscle clenching, walking, and cycling) reduce cessation barriers, such as cravings [4], withdrawal symptoms [5–12], perceived stress [7, 8], irritability and tension [7, 8], weight gain [6], and trouble concentrating [7, 8], and improve quitting self-efficacy [7, 8]. Consistent with these findings, prior smoking cessation trials combined with exercise [13–16] improved end-of-treatment smoking cessation rates, but only one study demonstrated that physical activity enhanced long-term (12-month) smoking abstinence [15].

Although previous studies [8, 14–16] have expanded the current understanding of the relationship between physical activity and smoking cessation, several limitations in methodology and intervention design have been reported. Methodological limitations include small sample sizes [16–26], the absence of men [8, 13, 14, 20, 22–31], and inadequate comparison groups. Prior trials have also reported poor adherence to and sustainability of the prescribed physical activity intervention. Moreover, there was considerable variation across studies in frequency and type of cessation treatment and physical activity logistics, such as initiation relative to quit date and frequency and intensity of exercise prescription [10]. Therefore, the most effective physical activity strategies and recommendations to enhance cessation and abstinence are yet to be established.

Previous efficacy studies have focused primarily on intensive, highly structured, supervised activity programs delivered for a short duration in a research setting [14–16, 18–22, 28, 29, 32, 33]. While this “one size fits all” approach is advantageous to evaluating effectiveness of a particular intervention, it may limit program access and participant self-efficacy for initiating and maintaining an active lifestyle, thereby minimizing the potential benefits physical activity may provide for long-term smoking cessation in community settings [34]. In fact, cessation programs have not typically provided the resources or skills needed to encourage individuals to adopt and maintain long-term adherence to physical activity goals [10]. Moreover, sedentary adults are more adherent to and prefer moderate-level activity instead of vigorous-level activity when starting an exercise program [35–38]. These considerations indicate that it is important to consider the potential influence that physical activity type, intensity, and setting may contribute to immediate and long-term cessation rates.

LEAP was designed to evaluate the efficacy of physical activity programming for smoking cessation using an extended treatment approach (one year), involving structured, moderate-intensity physical activity delivered in convenient and accessible community-based facilities (YMCAs), and recommendations for leisure time and “lifestyle” activities via individual fitness instruction and cognitive-behavioral skills training. Physical activity programming was integrated as an adjunct to standard “best practice” smoking cessation treatment (behavioral counseling and nicotine replacement) and was compared to a wellness intervention that was matched on contact frequency. We hypothesized that the physical activity intervention would produce significantly better smoking quit rates than the wellness comparison group through 12 months of follow-up.

## 2. Methods

### 2.1. Study Design

The LEAP study is a two-group, parallel-arm randomized controlled trial. All participants received a standard smoking cessation intervention (behavioral counseling plus nicotine patch) and were randomized to either an adjunctive physical activity intervention or a contact frequency-matched wellness comparison intervention. The primary outcome was biochemically verified prolonged smoking abstinence at 7 weeks, 6 months, and 1 year. Secondary outcomes were biochemically verified 7-day point prevalent abstinence (PPA) and changes in leisure-time physical activity and strength training at 7 weeks, 6 months, and 1 year. Adherence to treatment, treatment implementation fidelity, retention at follow-up, safety, and acceptability of the interventions were also documented.

Details of the LEAP trial design have been reported elsewhere [39]. Protocols and consent documents were approved by The University of Memphis and The University of Tennessee Health Science Center Institutional Review Boards and reviewed by an independent Data and Safety Monitoring Board. The trial was registered at www.ClinicalTrials.gov (identifier NCT00403312).

### 2.2. Study Participants

Participants included adults 18 to 65 years of age who smoked at least five cigarettes per day for one or more years and were interested in quitting. To be eligible, individuals needed to be able to speak/read English and were required to be sedentary or minimally active for the past six months, defined as engaging in ≤ three days per week of 30 minutes of moderate-intensity leisure-time physical activity (equivalent to brisk walking) and ≤ one day per week of 30 minutes of vigorous-intensity physical activity (equivalent to running), as measured by two brief items created for this study. Prior to randomization, participants completed a medical screen to ensure they were healthy enough to engage in physical activity, as described below. Study exclusionary criteria included inability to understand consent procedures, contraindications to nicotine replacement therapy (NRT) use (known contraindication or sensitivity to NRT, or currently pregnant, lactating, or intending to become pregnant, recent history of a cardiac event or procedure), history of a serious illness that might limit longevity or ability to participate in the study (e.g., significant renal disease, liver disease, cancer with life expectancy less than one year, or current substance abuse), and any of several health conditions that might be contraindications for initiating a physical activity program such as extremely elevated blood pressure, positive exercise tolerance test (ETT), uncontrolled arrhythmia or hyperthyroidism, symptomatic peripheral artery disease, 2nd or 3rd degree AV block on EKG, or congestive heart failure.

### 2.3. Recruitment and Screening

Participants were recruited via paid advertisements and public service announcements in local newspapers and on radio and television, free university media including telephone “on hold” announcements and stories in employee newsletters, physician referral, and “word of mouth.” Those interested contacted the project office and completed a brief telephone prescreen to determine whether they met the basic study requirements based on age, smoking status, physical activity level, health status, plans to remain in the area for the next year, and current pregnancy or plans to attempt pregnancy.

Once initially approved, potential participants were scheduled for the first of two in-person screening visits. During the first screening, study procedures, requirements, potential benefits, and risks were explained; informed consent was obtained; and demographic information and several self-report instruments were completed. The second screening consisted of a medical history and physical examination by a study physician because smoking cessation treatment included NRT (in both groups) and one intervention condition involved physical activity. The examination included an assessment of vital signs (blood pressure, pulse, and respiratory rate) and an electrocardiogram. A urine pregnancy test was given to any person who identified as female and indicated there was a possibility of being pregnant. All participants then underwent a maximal, symptom-limited exercise tolerance test (ETT) to screen for occult coronary artery disease. Individuals with positive ETT results were deemed ineligible and were referred to their personal physician for follow-up. Positive ETT results included ≥1 mm S-T segment depression or elevation in one or more leads during maximum exercise or during the recovery period, angina pectoris during exercise, arrhythmia, syncope, or an abnormal blood pressure response to exercise such as hypotension or severe hypertension.

Once participants met eligibility requirements as determined by the study physician, they attended a randomization visit where they completed final laboratory measures (height, weight, body fat, and blood pressure) and a 7-day physical activity recall [37]. After completing all measures, the study coordinator contacted the study biostatistician, who was not involved in assessment or intervention delivery, and who randomly assigned the participant using a 1 : 1 ratio to either the physical activity or wellness condition via a computer-generated uniform random number sequence.

### 2.4. Interventions

#### 2.4.1. Intervention Overview

Participants in both treatment conditions received behavioral smoking cessation counseling combined with NRT in the form of the transdermal nicotine patch. All behavioral interventions (smoking cessation, physical activity, and wellness) were informed by the social cognitive theory (SCT) [40] to enhance self-efficacy and address mood, perceived benefits of engaging in the behavior (outcome expectations), and barriers to achieving behavioral goals, which are personal factors and important cognitive determinants of changes in smoking, physical activity, and wellness behaviors [41–49]. In addition, and grounded in the self-regulation model of behavior change [50], participants were taught to use self-monitoring, self-evaluation, and self-reinforcement through goal setting, self-talk, and problem solving to enhance their ability to quit smoking, integrate more physical activity into their daily lives, and improve wellness behaviors. The details of the interventions can be found elsewhere [39].

#### 2.4.2. Intervention Procedures

Participants assigned to the physical activity condition received a combined smoking cessation/physical activity intervention which included 16 face-to-face physical activity/cessation sessions, 11 telephone activity/cessation sessions, and 11 supportive mailings, for a total of 38 intervention contacts. One-year YMCA memberships were provided by the study where all in-person meetings were conducted, with the exception of the seven-week and six-month visits, which were conducted in a university office. Participants in the wellness condition received the same smoking cessation intervention plus a general wellness program which included eight face-to-face wellness/cessation sessions, 12 telephone wellness/cessation sessions, and 18 follow-up mailings, for a total of 38 intervention contacts. Participants in the wellness condition received incentives during their face-to-face visits which included a t-shirt, movie passes, stress ball, notepad and pen, first-aid kit, backpack, cookbook, shower gift pack, and a $10 gift card. Intervention for the wellness condition was delivered in a university office. For both conditions, face-to-face sessions lasted 60-75 minutes during the initial four weeks of the program and 60 minutes subsequently. Phone sessions lasted approximately 20 minutes. The interventions were sequenced in both conditions, with the first four face-to-face sessions focusing on either physical activity or wellness, and the smoking cessation intervention introduced during the fifth face-to-face session.

All sessions were delivered by bachelor-level health and fitness instructors (HFI) with backgrounds in exercise science or health promotion who were cross-trained to deliver both interventions. The HFIs were trained on each intervention and supervised weekly by project coinvestigators. All face-to-face intervention sessions were audiotaped, and 25% were randomly selected, with 4–5 audiotapes reviewed by investigators, not involved in treatment delivery, discussed at each weekly meeting, and reviewed with HFIs weekly.

#### 2.4.3. Smoking Cessation Intervention

The standard smoking cessation intervention provided for both treatment conditions included NRT and behavioral counseling. Participants were provided with six weeks of transdermal nicotine, commencing on the date of their quit attempt. Patch dosage was initially established from the number of cigarettes smoked at baseline, and then a gradual dose tapering strategy was used. The behavioral cessation counseling involved four primary phases: (1) preparing to quit; (2) going through the quitting process; (3) maintaining short-term smoking abstinence; and (4) relapse prevention and long-term maintenance. The cessation intervention was delivered primarily during four face-to-face sessions, occurring one week before and one and three weeks after the scheduled quit day, with brief follow-ups occurring during other in-person and telephone sessions.

#### 2.4.4. Physical Activity Intervention

The physical activity intervention included the following components: (1) developing an individualized exercise plan for each participant based on personal characteristics and preferences; (2) targeting, as a goal, the Centers for Disease Control and Prevention/American College of Sports Medicine recommended levels of activity (150 minutes of moderate-to-vigorous activity each week [51]); (3) incorporating moderate as well as vigorous intensity activity and strength training; (4) encouraging planned exercise sessions, “lifestyle activity” (activity that is generally not planned and occurs in the context of working, traveling, raising children, etc., such as housework, yard work, and dog-walking), and short bouts (≥ 10 minutes) of structured or lifestyle activity, as needed, to manage urges to smoke and other withdrawal symptoms; (5) providing both supervised activity sessions and behavioral counseling to increase physical activity self-management skills (adapted from *Active Living Everyday*) [52]; (6) improving accessibility by offering the program at 10 convenient YMCA locations in the community; and (7) providing supportive contact during the maintenance phase (through 12-months) to boost adherence.

#### 2.4.5. Wellness Intervention

Participants in the comparison condition received an individually tailored general wellness curriculum that covered the multiple dimensions of wellness and how to achieve balance in one's life. Topics included the continuum of health and the components of wellness; primary risk factors for poor health; the components of wellness and the importance of a balanced lifestyle; physical wellness emphasizing awareness of prevention strategies and the importance of early detection and treatment; social wellness; awareness of injury risk; the importance of good nutrition, reading food labels, and serving sizes; making healthy food choices and eating for optimal wellness; causes and ways to avoid and/or minimize injury, reducing stress; and ways to improve emotional health.

### 2.5. Measurements

Data collection was conducted at baseline and 7 weeks, 6 months, and 12 months after randomization. All assessments were taken by the research staff not involved in the intervention who were trained using a common, standardized protocol. Measurement staff were periodically monitored and received additional training as needed. A detailed description of measurement variables can be found in Table 1 or the baseline article [39].

#### 2.5.1. Baseline Characteristics

Sociodemographic variables (gender, race, ethnicity, marital status, education, and age), psychosocial functioning (depressive symptoms, perceived stress, and social support), tobacco-related variables (smoking history, dependence, nicotine withdrawal symptoms, self-efficacy for quitting smoking, confidence, motivation, and support to quit), and physical activity variables (physical activity stages of change, positive and negative physical activity attitudes, and self-efficacy for physical activity) were collected via a self-report questionnaire.

For psychosocial functioning, depressive symptoms were measured with the Center for Epidemiologic Studies-Depressed Mood Scale (CES-D) [53]. Participants who scored at or above the established cut-off for elevated depressive symptoms (≥16) were contacted by a trained professional and offered a mental health referral. Perceived stress was measured using the 14-item perceived stress scale (PSS) [54–56]. General social support was measured using a revised version of the 12-item perceived social support (PSS) scale [56].

For the tobacco-related variables, smoking history and patterns were measured and included number of years smoked and number of previous quit attempts of ≥24 hours. Nicotine dependence was assessed using the Fagerström test of nicotine dependence (FTND) [57]. To assess nicotine withdrawal symptoms, we used the Minnesota withdrawal scale (MWS) [58, 59]. Self-efficacy for quitting smoking and staying abstinent was assessed using the 9-item self-efficacy/temptation (short form) [60]. There is an overall total score and three sub-scale scores. Support for quitting smoking was measured using the partner interaction questionnaire (PIQ) which included 20 items that resulted in a score for positive and negative behaviors [61]. Confidence in staying quit and motivation to quit were both assessed with a one-item question each measured on a 10-point Likert-type scale with higher scores indicating greater confidence and motivation.

Positive and negative physical activity attitudes were measured by a 10-item decisional balance inventory that assesses the perceived pros and cons of engaging in exercise [62]. Self-efficacy for exercise was measured using the exercise self-efficacy scale [63, 64]. The six-item version was used to assess how confident the participants were in their ability to exercise in response to common barriers to physical activity, with a higher total score indicating higher self-efficacy for exercise.

#### 2.5.2. Primary Endpoint

The primary endpoint was prolonged abstinence at the end of treatment (7 weeks) and 6- and 12-month postcessation, defined as a self-report of no smoking throughout the follow-up period after allowing for a two-week “grace period” after the quit attempt [65] and an exhaled carbon monoxide (CO) level of <10 ppm. If a participant self-reported being abstinent but had an elevated CO level (≥10 ppm), salivary cotinine was assessed as a secondary biomarker of tobacco use. Assuming a power of .80 and a two-tailed level of significance of alpha = 0.05 and beta = 0.20, we set our target enrollment at 400 participants (200 per group) to detect 12-month prolonged abstinence rates of 25% vs. 38% in the comparison vs. treatment conditions, respectively.

#### 2.5.3. Secondary Endpoints

Seven-day point prevalent abstinence (PPA) was assessed, defined as a self-report of no smoking during the past seven days at a particular follow-up period with no correction for previous or subsequent smoking status, with an expired carbon monoxide level <10 ppm. As with prolonged abstinence, a salivary cotinine level was considered to indicate smoking if the participant reported abstinence but had elevated CO.

To assess leisure-time physical activity and strength training, we used the seven-day physical activity recall [66]. The PAR is a semistructured interview that estimates an individual's time spent in physical activity, as well as strength and flexibility activities, in the past seven days. The compendium of physical activities [67] was then used to code MET intensity values for activities reported by participants. As recommended by Sallis et al. [66], occupational-related activity was differentiated from leisure time activity (LTPA), and considering that participants were prescreened for eligibility based on LTPA, analyses focused on total minutes per week of LTPA and strength training.

All PAR interviews were audiotaped. A random 20% of these were periodically reviewed by project coordinators to determine fidelity in administering (e.g., “explains intensity guidelines (walk = moderate, run = very hard)”) and scoring (e.g., “consistently and correctly records type of activity along with duration”). The overall mean administration and scoring competency rates across interviewers, participants, and follow-up assessments were 98.5% and 95.8%, respectively. Lack of fidelity was due mainly to failure by interviewers to probe adequately to determine intensity levels. In addition, audiotapes and PAR scoring sheets were reviewed as needed to clarify levels of intensity. As LEAP aimed to increase LTPA, only LTPA and strength training data were analyzed and reported.

#### 2.5.4. Treatment Implementation, Adherence, Retention, and Participant Satisfaction

Several indicators were used to assess treatment implementation, adherence, retention, and satisfaction. All face-to-face intervention sessions were audiotaped with a random selection of 10% being reviewed by the research investigators. The reviewer completed a criteria checklist for the accuracy of intervention implementation. Intervention adherence was measured by documenting face-to-face group attendance, phone contact adherence, and number of mailings sent. Retention rates were measured by the percentage of participants who completed the 7-week, 6-month, and 1-year follow-up visits. Lastly, participants completed a brief questionnaire at baseline and end-of-treatment to assess program satisfaction.

### 2.6. Statistical Analysis

All analyses were conducted using SPSS version 27 [68] as well as SAS version 9.4 [69]. Between-group differences in baseline characteristics, indices of treatment implementation, adherence, retention, treatment perception, and abstinence rates were assessed using chi-square analyses, *t-*test, or one-way analysis of variance (ANOVA) as appropriate. No significant differences were noted. Primary outcome analysis was based on the intent-to-treat principle, with all individuals analyzed as part of the treatment group to which they were assigned regardless of adherence, and individuals with missing outcome data or self-report abstinence not confirmed by CO or salivary cotinine at any follow-up point classified as not quitting. In addition, as the assumption that those lost to follow-up are still smoking may not necessarily provide a conservative estimate of the intervention effect [70], we reanalyzed outcomes for those 324 participants (83% of our population, 166 in the physical activity arm, and 158 in the wellness) who completed 12-month follow-up.

Trends in LTPA and strength training were examined using linear mixed modeling in SPSS. This procedure allowed us to test changes in LTPA outcomes over time, between treatment groups, and group-by-time effects.

To examine multivariable predictors of prolonged abstinence through 12-months, a generalized estimate equation (GEE) analysis for binary data was performed using GENLIN in SPSS. This procedure allowed us to consider observations between participants as independent and observations within participants as correlated. All baseline and time-varying variables from Table 2 were used in the analyses.

## 3. Results

### 3.1. Study Population

**Figure 1:**
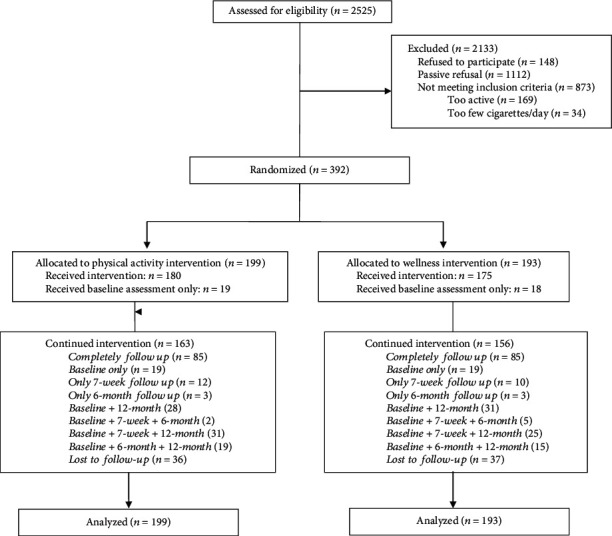
Enrollment and retention.

Recruitment began in July 2004 and ended in September 2007. Of the 2,525 respondents to our recruitment efforts, 1413 agreed to be screened, and 403 of these qualified to be in the study after completing the medical screen. Of these 403 qualified individuals, four were ineligible at randomization and seven passively refused (Figure 1), resulting in a total of 392 who were enrolled and randomized into the physical activity group (*n* = 199) or the wellness group (*n* = 193). Participants had an average age of 44.6 (10.2) years with 67% identifying as female, 67% Caucasian, and 31% African American. Twenty-six percent had at least some college education. Overall, participants smoked an average of 21 (9.5) cigarettes per day and had been smoking for 23.8 (10.7) years with only 11% having never made a quit attempt. The mean values and distributions of the major demographic, physical, psychosocial, and physical activity measures at baseline were similar for the two intervention arms (Table 2).

### 3.2. Primary Endpoint

#### 3.2.1. Smoking Cessation

Intent-to-treat analyses, using penalized imputation, indicated that biochemically confirmed prolonged abstinence for the physical activity and wellness groups, respectively, was 19.6% and 25.4% at 7 weeks, 15.1% and 16.1% at 6 months, and 14.1% and 17.1% at 12 months. Between-group differences were not significant at any follow-up (all *P* values >0.18); see Table 3 (part a).

In addition, restricting analyses to those participants who provided follow-up data at a particular follow-up point (i.e., complete case analysis), the proportion of participants in the physical activity and wellness groups who met criteria for prolonged abstinence was 31.1% and 37.8%, respectively, at 7 weeks, 27.5% and 29.6%, respectively, at 6 months, and 17.2% and 20.3%, respectively, at 12 months. Between-group differences were not significant at any follow-up (all *P* values >0.24); see Table 3 (part b).

### 3.3. Secondary Endpoints

#### 3.3.1. 7-Day Point Prevalent Abstinence

Biochemically confirmed seven-day point prevalent abstinence rates for the physical activity and wellness groups, respectively, were 24.6% and 31.6% at 7 weeks, 19.6% and 21.8% at 6 months, and 22.6% and 23.3% at 12 months. Between-group differences were not significant at any follow-up (all *P* values> .14); see Table 3 (part a).

In addition, similar analyses were done with only the participants who provided follow-up data at a particular follow-up point. Seven-day point prevalent abstinence rates for the PA and wellness groups were 26.4% and 27.2%, respectively, at 12 months, 36.1% and 39.6% at 6 months, and 38.6% and 49.6% at 7 weeks. Between-group differences were not significant at any follow-up (all *P* values >0.08); see Table 3 (part b).

### 3.4. Physical Activity Levels

Linear mixed modeling examining the total number of minutes spent in strength training plus LTPA by group (physical activity or wellness), time of assessment, and group by time revealed an interaction between time and group in strength training at 7 weeks (*P* = 0.04). Minutes spent in strength training plus LTPA were greater in the physical activity treatment group at 7 weeks only; see Table 4.

### 3.5. Predictors of Prolonged Cessation at 12 Months

GEE was used to examine the relationship between predictors and prolonged abstinence, as well as the relationship between time and smoking cessation. All variables from Table 2, including treatment condition, were initially subjected to bivariable GEE analyses. Those variables that were significant at a *P* < .10 were entered into the multivariable model. Those included baseline-only variables: age (*P* = 003), gender (*P* = .093), quit attempts (*P* = .052), FTND score (*P* = .051), and number of years smoked (*P* = .008). Change over time variables included were CES-D (*P* = .006), levels of perceived social support (*P* ≤ .001), motivation to quit (*P* = .045), confidence to remain quit (*P* = .001), temptation to smoke by habit (*P* = .001) mean positive social situations (*P* ≤ .001) or negative social situations (*P* = .001), and support to quit smoking (*P* = .031). The final model (see Table 5) indicated that greater perceived social support from family and friends, fewer years smoked, as well as less temptation to smoke due to habit, positive affect/social situations, and negative affect situations, were associated with a greater likelihood of prolonged abstinence over 12 months. Results also specified that over time (referent = 12 months), participants' likelihood of quitting decreased (odds ratio at 7 weeks = 4.435; *P* ≤ .001 and odds ratio at 6 months = 2.752; *P* ≤ .001).

### 3.6. Treatment Implementation, Satisfaction, Adherence, and Retention

#### 3.6.1. Implementation

Analysis indicated that the overall intervention was delivered accurately in ≥ than 90% of sessions in both arms of the study. The most common examples of inaccurate delivery were not adequately addressing follow-up action plans or self-monitoring of smoking. Analysis of variance (ANOVA) indicated that there were no significant differences between treatment arms (*P* = 0.89) or among the health educators (*P* = 0.29) in intervention delivery. The accuracy of intervention delivery was ≥90% for all health educators.

#### 3.6.2. Satisfaction

The majority of participants in both the PA and wellness conditions found the intervention to be helpful in helping to quit smoking (89.5% and 80.0%, respectively (*P* = 0.01)). Time commitment to the program was considered to be “slight” or “not at all a burden” (93.3 and 89.4%, respectively (*P* = 0.36)). Most participants agreed “strongly” that they would recommend the program to a friend (74.8% and 71.7%, respectively (*P* = 0.04)). In addition, participants agreed that their health educators were knowledgeable about smoking cessation (98.2% and 91.9%, respectively (*P* = 0.33)), skilled in helping change their smoking behavior (82.8% and 73.1%, respectively (*P* = 0.02)), and cared about his/her success in the program (95.1% and 90%, respectively (*P* = 0.27)). Of the participants who wrote comments on the 12-month process evaluation (*n* = 129), the most frequent complaints registered by participants in both groups were wanting more face-to-face sessions (14.0%) and too many questionnaires (10.9%). The most common criticism in the physical activity condition (*n* = 59) was too few face-to-face sessions (15.3%). Among wellness participants (*n* = 70), the most frequently voiced complaint was not being in the physical activity arm (14.3%).

#### 3.6.3. Adherence

Face-to-face session attendance over the year averaged 62.7% for the physical activity group (10.1 of 16 sessions) and 71.8% (5.9 of 8 sessions) for the wellness group (*P* ≤ .001). There were no significant differences between the groups on phone contact adherence, with the physical activity group averaging 53.4% (6.0 of 11 calls) and the wellness group averaging 54.5% (6.8 of 12 calls) (*P* = .778). There were no significant differences (*P* = .884) between the groups in the number of mailings sent (physical activity = 84.9% and wellness = 84.4%).

#### 3.6.4. Retention

At the 7-week follow-up visit, 66.1% (259/392) of the baseline sample were available for assessment, 55.4% (217/392) were available at the 6-month follow-up visit, and 82.7% (324/392) at the 1-year follow-up visit (Figure 1). There were no significant group differences in retention at any follow-up (*P* values >0.79).

#### 3.6.5. Safety

Only 3.8% (*n* = 15) of all participants reported study-related symptomatology. Three participants in the physical activity condition reported muscular/skeletal pain from exercising, and 8 reported patch-related issues (skin irritation at the patch site, *n* = 5; disturbed sleep, *n* = 2; and nausea, *n* = 1). Four participants in the wellness condition reported patch-related issues (skin irritation at the patch site, *n* = 1; tachycardia, *n* = 2; and dizziness *n* = 1).

## 4. Discussion

The purpose of this study was to determine whether flexible, individually tailored physical activity programming delivered in a community setting would improve long-term smoking cessation rates compared to a contact frequency-matched wellness-focused comparison intervention. Physical activity and wellness programs were delivered as adjunctive care with standard behavioral/pharmacological smoking cessation treatment. Contrary to our hypothesis, the physical activity intervention did not improve cessation outcomes compared to the wellness intervention, although both groups achieved respectable long-term cessation rates (one year after the quit attempt). In addition, 7-day point prevalence of abstinence was 22.6% in the physical activity and 23.3% in the wellness groups. In fact, the long-term quit rates we achieved are higher than what has been reported by other smoking cessation trials that utilized adjunctive physical activity programming. For example, the most successful trial to date [15] achieved a 12% continuous abstinence rate at one year using standardized, supervised vigorous activity. Similarly, a program using standardized, supervised moderate activity achieved 12% continuous abstinence at one year [27], and moderate activity delivered in a community setting (YMCAs) achieved 7% continuous abstinence at one year [31]. While intervention and sample differences between these previous studies and our own, as described in the Introduction, preclude direct comparison, our relatively high quit rates are promising. This combined with the findings that indicated that prolonged abstinence is greater for those who have social support, have been smoking for fewer years, and are exposed to fewer temptation can help tailor effective intervention efforts.

One contributor to the lack of a treatment effect may be that the two groups received identical behavioral and pharmacological cessation support, along with intensive adjunctive care that emphasized overall health improvement (physical activity or general wellness). Satisfaction was slightly higher on some measures in the physical activity group than the wellness group, but the vast majority of participants in both groups were satisfied with the treatment they received; likewise, adherence was reasonable in both groups (e.g., both groups received about two-thirds of scheduled face-to-face contacts and half of phone contacts, which is similar to other smoking/physical activity interventions) [17, 27, 31]. While physical activity adherence was suboptimal in the physical activity group, increases were also observed in the wellness group, suggesting that the wellness program, although it did not emphasize physical activity, may have motivated participants to increase their activity. Enrolling a third group that received minimal treatment would have allowed us to tease apart the specific effects of the physical activity intervention from the general effects of intensive contact but would have added considerable time and cost to the study.

The literature on the efficacy of physical activity to promote smoking cessation is mixed [10]. The best evidence to date that physical activity boosts long-term abstinence comes from a study by Marcus et al. [15] which utilized highly structured, group-based vigorous activity delivered in a laboratory setting. The dissemination potential and sustainability of this approach, however, appeared to be very limited, as most eligible individuals who smoke elected not to participate, long-term quit rates were fairly low (12% at one year), and only 10% of these initially sedentary participants continued to engage in regular exercise 12 months following treatment. To overcome these limitations, our study elected to individualize physical activity programming. This was done by providing tailored exercise prescriptions that included participant-preferenced activities, emphasizing a combination of moderate, vigorous, and “lifestyle” activity, making exercise as convenient as possible by offering membership and training at a number of YMCAs, and extending the intervention for one year to encourage long-term adoption of new behaviors. Despite this flexible approach, adoption of physical activity was suboptimal, increasing in the physical activity group by only 51 minutes per week at the first follow-up (7 weeks after the intervention commenced) and returning to near baseline levels (138.3 vs. 155.6 minutes per week) by the 6-month follow-up. The only significant difference in physical activity between the physical activity and wellness groups in this study was found at the 7-week follow-up when the physical activity group reported more time spent in strength training per week than the wellness group (29.7 minutes vs. 12.9 minutes, respectively). These results point to the significant challenge of facilitating the adoption and maintenance of long-term physical activity among previously sedentary adults who smoke. Since participation in physical activity appeared to decrease as participants transitioned toward becoming more self-reliant and reducing the frequency of individual sessions with trainers, it may be necessary to extend the duration of professional contact to sustain activity levels and cessation. Such intensive intervention is unlikely to be acceptable to many smokers who want to quit, but our results indicate that some individuals who smoke are likely to find this approach helpful. Dissemination potential could be considerable if community organizations such as the YMCA embedded smoking cessation counseling into personal training services for members, as we did in the present study.

Several study limitations must be noted including design factors, adherence, instrumentation, and retention. First, a limitation of the study design was a lack of a no- or minimal-treatment control group which would have helped delineate treatment results. Second, because of the intensity of the intervention (multiple behavioral counseling sessions and nicotine patch), we did not recruit very light smokers (those who smoked fewer than 5 cigarettes/day) who typically are not prescribed pharmacological treatment [2] and are less likely to participate in cessation treatment [71]; therefore, our results do not generalize to this group. Second, face-to-face session attendance for both groups was suboptimal, averaging 63% and 72% for the physical activity and wellness groups, respectively. This lower adherence in the physical activity group may reflect the increased burden (16 vs. 8 scheduled face-to-face sessions for physical activity vs. wellness). Treatment effects may have been better had adherence to physical activity been better. While the level of physical activity adherence we achieved is similar to that of other trials [72], it indicates the continuing challenge of encouraging long-term maintenance of physical activity in previously sedentary individuals who smoke. On the plus side, the vast majority of participants in the physical activity group perceived the intervention to have helped them quit smoking and would recommend the program to others who are trying to quit smoking; further, physical activity participants were generally more satisfied than wellness participants with the extent to which adjunctive treatment (physical activity or wellness) helped their cessation effort.

Another limitation is that we relied on self-report to assess physical activity change. Although the PAR is a well-accepted physical activity assessment, it is less robust than objective methods of measuring moderate physical activity and is influenced by external factors, such as social desirability and memory bias [73, 74]. The study could be strengthened by the use of objective measurement of physical activity and with participant tracking logs to support the findings.

Retention was another limitation in that it was not ideal. While 84% of participants returned for the 1-year follow-up, only 66% and 55% completed the 7-week and 6-month assessments, respectively. Retention rates did not differ significantly by the treatment group, reducing the risk of differential misclassification. We used penalized imputation in our intention-to-treat outcome analyses, but there is evidence from other cessation trials that this may not necessarily be a conservative approach to estimating treatment effects [75]. To address this potential limitation, we conducted sensitivity analyses, including a GEE model that used all available data from all participants and a logistic analysis that was restricted to only participants who provided follow-up data, and all results were similar.

A final limitation is that the data were collected several years ago, and the results may not generalize to individuals who are currently making a quit attempt. This does not seem likely, however, since our sample characteristics are very similar to those of other most recent cessation trials that utilized physical activity and recruited from the general community [76–78].

Despite these limitations, this study found that a flexible, individualized, community-based physical activity program was acceptable to individuals undergoing a smoking quit attempt and led to comparable quit rates in relation to the extant literature, although these rates were not higher than what was achieved using a general wellness program. Given the continuing burden of smoking, the high comorbidity of smoking and a sedentary lifestyle, and evidence from several studies that physical activity can aid quit attempts, more work is needed to determine how to best encourage the adoption and maintenance of active lifestyles among those who smoke and want to quit.

## Figures and Tables

**Table 1:** Description of self-report measures.

Measure [ref. number]	Description	Calculation
*Psychosocial variables*		
Center for Epidemiologic Studies-Depressed Mood (CES-D) (20 items) [53]	Assesses symptoms of depressed mood	Participants were asked to rate their endorsement of 20 items on a Likert-type scale ranging from 1 (rarely or none of the time) to 4 (most or all of the time). Four of the items were reverse-scored. The questionnaire was summed. Lower scores are associated with less depressive symptomatology.
Perceived stress scale (PSS) (14 items) [54, 55]	Used to assess the perceived level of stress the participant may be experiencing.	Participants were asked to rate how often they felt a certain way using a 5-point Likert-type scale ranging from 0 (never) to 4 (very often). Positive items (7) were reversed scored. Scores were summed. Lower scores suggest lower levels of stress.
Perceived social support scale [56]	Assesses general social support	Participants were asked to rate their agreement for each situation on a Likert-type scale ranging from 1 (very strongly disagree) to 7 (very strongly agree). Scores were totaled. Higher scores indicated greater social support.

*Smoking variables*		
Fagerstrom test of nicotine dependence (FTND) (6 items) [57]	Assessment of the degree of nicotine dependence	The 6 items are coded and totaled. Scores range from 0 to 10. Higher totals indicate greater dependence.
Minnesota withdrawal scale (MWS) (9 items) [58, 59]	Assessment of nicotine withdrawal symptoms	Participants rated the degree to which they experienced the 9 symptoms on a scale from 0 (none) to 4 (severe). A mean is extracted. Higher numbers indicate greater symptomatology.
Self-efficacy for quitting smoking/temptation to smoke (9 items) [60]	Assesses temptation to smoke in 3 distinct areas (habit, positive, and negative social situations).	Participants rate the degree to which they are tempted to smoke on the 9 items (3 items for each category). Scores range from 0 (not tempted at all) to 4 (extremely tempted). Scores are summed. Higher scores indicate a higher level of temptation.
Partner interaction questionnaire (20 items) [61]	Assesses the degree of positive or negative support, given to the participants' smoking cessation efforts, from a spouse, partner, other	Participants were asked to rate their agreement for each item on a Likert-type scale ranging from 0 (never) to 4 (very often). Scores were summed for each subscale (positive or negative). Higher scores are associated with greater positive/negative support
Confidence in staying quit permanently (1 item)	Assesses the level of confidence that the participant has in remaining free from smoking.	Confidence levels are rated on a 10-point Likert-type scale with 0 indicating not at all confident and 10 indicating extremely confident. A higher score is associated with greater confidence.
Motivated to quit smoking (1 item)	Assesses motivation to quit smoking.	Assesses motivation to quit smoking using a 10-point Likert-type scale 0 indicating not at all motivated to 10 indicative of being extremely motivated. A higher score is associated with greater motivation.

*Physical activity variables*		
Physical activity stage of change (5 items) [64]	Assesses stages of change in exercise.	Participants selected the statement that most accurately described.
Physical activity decisional balance (10 items) [62]	Assesses positive and negative aspects of exercise.	Participants rated the importance of aspects of exercise on a Likert-type scale from 1 (not important) to 5 (very important). Two subscales (sum of 5 positive and 5 negative items) were created. Higher scores indicate greater positive/negative attitudes toward exercise.
Self-efficacy for physical activity (6 items) [63]	Assesses confidence in continuing to engage in exercise when life events (e.g., rain) intrude.	Participants rated how confident they were to continue exercising on a Likert-type scale from 1 (not at all confident) to 5 (completely confident). A mean score was used for analyses. Higher scores suggest greater confidence levels.
		Their current and recent activity levels and future intentions representing the stages of exercise behavior change.

**Table 2:** Baseline characteristics of LEAP participants (mean (SD)or (%)).

Variable *n*	Physical activity 199	Wellness 193
*Demographic measures*		
Age (yrs)	44.6 (9.9)	44.6 (10.4)
Gender, *n* (%)		
Male	70 (35.2)	79 (40.9)
Ethnicity, *n* (%)		
Hispanic	0 (0.0)	4 (2.1)
Not Hispanic	198 (99.5)	186 (96.4)
Missing	1 (0.5)	3 (1.6)
Race, *n* (%)		
White	131 (65.8)	130 (67.4)
AA	64 (32.2)	57 (29.5)
Other	4 (2.0)	6 (3.1)
Marital, *n* (%)		
Never	28 (14.1)	34 (17.6)
Married/cohabiting	96 (48.2)	102 (52.9)
Divorced/Sep.	67 (33.7)	53 (27.5)
Widowed	8 (4.0)	4 (2.1)
Education, completed college *n* (%)		
College graduate	43 (21.6)	38 (19.7)
Or >	11 (5.5)	11 (5.7)
*Psychosocial*		
Depressive symptoms (CES-D)	11.3 (9.4)	12.7 (10.4)
Perceived stress	22.1 (7.5)	22.4 (8.9)
Perceived social support	66.9 (14.5)	65.6 (14.7)
*Tobacco use*		
Total years smoking	23.6 (9.8)	24.0 (11.7)
No. quit attempts, *n* (%)		
Never	19 (9.6)	25 (13.0)
1-5 times	115 (58.4)	104 (53.9)
6-10 times	30 (15.2)	33 (17.1)
11-15 times	10 (5.1)	8 (4.1)
16 or more times	23 (11.7)	23 (11.9)
Fagerström test of nicotine dependence	4.7 (2.3)	5.1 (2.3)
Nicotine withdrawal symptoms	0.93 (.62)	1.02 (.74)
Self-efficacy for quitting smoking/temptation to smoke		
Habit	3.52 (.90)	3.60 (.87)
Positive affect/social situations	3.91 (.79)	3.86 (.87)
Negative affect situations	4.38 (.73)	4.24 (.75)
Support to quit (positive)	14.2 (11.4)	13.7 (10.6)
Support to quit (negative)	14.9 (10.8)	13.4 (11.2)
Confident can stay quit	8.1 (2.1)	8.0 (2.1)
Motivation to quit smoking	8.8 (1.5)	8.8 (1.3)
*Physical activity*		
Positive PA attitude	20.2 (4.3)	20.3 (4.4)
Negative PA attitude	6.4 (2.1)	6.6 (2.5)
Self-efficacy for PA	18.7 (5.9)	17.8 (5.7)

**Table:** (a) Cessation rates at endpoints (intent to treat)

	Seven weeks			Six months			Twelve months		
	PA (*n* = 199)	Wellness (*n* = 193)	(*P*)	PA (*n* = 199)	Wellness (*n* = 193)	(*P*)	PA (*n* = 199)	Wellness (*n* = 193)	(*P*)
Prolonged	19.6%	25.4%	(.18)	15.1%	16.6%	(.78)	14.1%	17.1%	(.49)
Seven day									
Point prevalent	24.6	31.6%	(.14)	19.6%	21.8%	(.62)	22.6%	23.3%	(.90)

**Table:** (b) Cessation rates at endpoints (participants attending follow-up)

	Seven weeks			Six months			Twelve months		
	PA (*n* = 132)	Wellness (*n* = 127)	(*P*)	PA (*n* = 109)	Wellness (*n* = 108)	(*P*)	PA (*n* = 166)	Wellness (*n* = 158)	(*P*)
Prolonged	31.1%	37.8%	(.24)	27.5%	29.6%	(.88)	17.2%	19.6%	(.67)
Seven day									
Point prevalent	38.6%	49.6%	(.08)	36.1%	39.6%	(.84)	26.4%	27.2%	(.94)

**Table 4:** Physical activity change scores by time.

Predictors	Estimates	CI	*P*	df
(Intercept)	38.29	-10.9-87.4	0.127	656.6
Time (7 weeks)	-31.33	-91.4-28.7	0.306	593.5
Time (6 months)	7.48	-514.2-66.2	0.802	425.1
PA Group	-18.6	-87.8-50.6	0.598	655.2
7 weeks^∗^PA	87.1	2.77-171.4	**0**.**043**	590.7
6 months^∗^PA	14.7	-68.3–97.7	0.728	421.9

**Table 5:** Predictors of prolonged smoking cessation from end of treatment to 12-month follow-up determined by generalized estimating equation (GEE) regression with available data.

	Odds ratio	95% OR	*P*
*Baseline variables*			
Age (yrs)	1.03	0.99-1.07	0.165
Gender			
Female	Ref.		
Male	0.72	0.39-1.36	0.314
Depression score (CES-D)	0.98	0.95-1.01	0.253
Perceived social support	1.02	1.00-1.04	0.005
Total years smoked	1.05	1.01-1.09	0.014
Number of quit attempts	0.98	0.76-1.26	0.853
Fagerstrom test of nicotine			
Dependence (FTND)	1.00	0.87-1.16	0.971
Motivation to quit smoking	0.84	0.68-1.04	0.114
Confidence for quitting smoking	1.00	0.86-1.63	0.994
Temptation to smoke			
Positive affect/social situation	0.61	0.43-0.88	0.008
Negative affect situations	0.49	0.36-0.68	≤0.001
Habitual/craving situation	0.62	0.40-0.94	0.026
Support to quit/positive	1.01	0.99-1.03	0.416
Nicotine withdrawal symptoms	1.61	0.99-2.64	0.057
Time 7 weeks	4.44	2.79-7.06	≤0.001
Time 6 months	2.75	1.83-4.14	≤0.001
Time 12 months	Ref.		

## Data Availability

The Lifestyle Enhancement Program data used to support the findings of this study are available from the corresponding author upon request.
